# An essential RNA element resides in a central region of hepatitis E virus ORF2

**DOI:** 10.1099/vir.0.051870-0

**Published:** 2013-07

**Authors:** Suzanne U. Emerson, Hanh T. Nguyen, Udana Torian, Karly Mather, Andrew E. Firth

**Affiliations:** 1 Molecular Hepatitis and Hepatitis Viruses Sections, Laboratory of Infectious Diseases, National Institute of Allergy and Infectious Diseases, National Institutes of Health, Bethesda, MD 20892, USA; 2 Division of Virology, Department of Pathology, University of Cambridge, Tennis Court Road, Cambridge CB2 1QP, U.K.

## Abstract

Hepatitis E virus (genus *Hepevirus*, family *Hepeviridae*) is one of the most important causes of acute hepatitis in adults, particularly among pregnant women, throughout Asia and Africa where mortality rates can be 20–30 %. Hepatitis E virus has a single-stranded positive-sense RNA genome that contains three translated ORFs. The two 3′ ORFs are translated from a subgenomic RNA. Functional RNA elements have been identified in and adjacent to the genomic 5′ and 3′ UTRs and in and around the intergenic region. Here we describe an additional RNA element that is located in a central region of ORF2. The RNA element is predicted to fold into two highly conserved stem–loop structures, ISL1 and ISL2. Mutations that disrupt the predicted structures, without altering the encoded amino acid sequence, result in a drastic reduction in capsid protein synthesis. This indicates that the RNA element plays an important role in one of the early steps of virus replication. The structures were further investigated using a replicon that expresses *Gaussia* luciferase in place of the capsid protein. Single mutations in ISL2 severely reduced luciferase expression, but a pair of compensatory mutations that were predicted to restore the ISL2 structure, restored luciferase expression to near-WT levels, thus lending experimental support to the predicted structure. Nonetheless the precise role of the ISL1+ISL2 element remains unknown.

## Introduction

Hepatitis E virus (HEV; reviewed by Jameel, 2011) is the causative agent of hepatitis E, an acute form of hepatitis that does not usually progress to chronicity. Hepatitis E can cause significant mortality due to liver failure, especially among pregnant women. HEV is associated with an estimated 271 000 deaths annually (Wierzba & Panzner, 2012). The virus is generally transmitted faecal–orally via contaminated water, but can also be acquired zoonotically from the ingestion of infected meat. It is especially prevalent throughout much of Asia, Africa and the Middle East. Currently the virus is classified into four genotypes of a single species, HEV, in the genus *Hepevirus* of the family *Hepeviridae*. These four genotypes and related sequences have been isolated from human hosts and/or other mammals including swine, rabbits and deer (Zhao *et al.*, 2009; Takahashi *et al.*, 2011). Related but highly divergent sequences have been isolated from chickens (avian HEV, ~45 % amino acid identity to human HEV; Huang *et al.*, 2004), rats and ferrets (~52 % amino acid identity to human HEV; Johne *et al.*, 2010; Raj *et al.*, 2012) and bats (~47 % amino acid identity to human HEV; Drexler *et al.*, 2012). An even more divergent HEV-like virus has been isolated from cutthroat trout (~25 % amino acid identity to human HEV; Batts *et al.*, 2011).

**Fig. 1.**
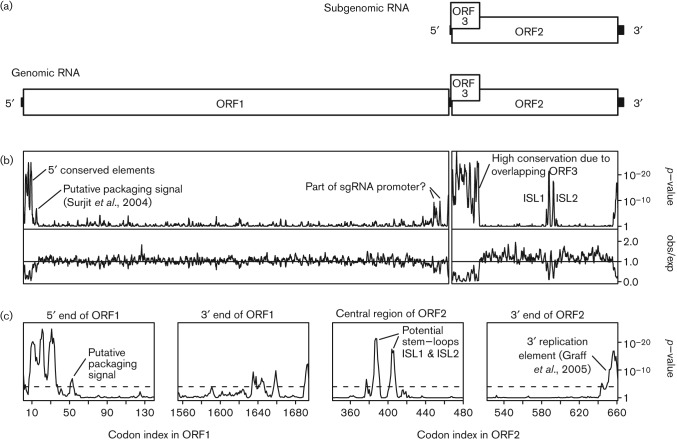
HEV genome organization. (a) Map of the ~7.2 kb genome. ORF1 is translated from the genomic RNA and encodes the replication protein domains including the RNA-dependent RNA polymerase. ORF2 and ORF3 are translated from a sgRNA. ORF2 encodes the capsid protein. (b) Conservation at synonymous sites in alignments of 185 ORF1 and 205 ORF2 nucleotide sequences, using a 5-codon sliding window. The putative packaging signal is according to Surjit *et al.* (2004). The lower panels show the ratio of the observed number of substitutions to the number expected under a null model of neutral evolution at synonymous sites, while the upper panels show the corresponding *P*-values. In order to map the conservation statistic onto the coordinates of a specific sequence in the alignment, all alignment columns with gaps in a chosen reference sequence (viz. NC_001434) were removed (note that the ORF2 alignment had no gaps in the reference sequence). Note, as expected, the extreme reduction in ORF2-frame synonymous substitutions in the region where ORF2 overlaps with ORF3. (c) Zoom-in of four regions that show particularly pronounced conservation at ORF1- or ORF2-frame synonymous sites. The dashed line indicates a *P* = 0.05 threshold after applying a rough correction for multiple testing (viz. 0.05/[2355 codons/5-codon window] ~ 1.1×10^−4^).

HEV has a single-stranded positive-sense RNA genome of ~7.2 kb. The genome contains three translated ORFs (Fig. 1a; reviewed by Ahmad *et al.*, 2011). ORF1 is translated from the genomic RNA and encodes the non-structural protein domains such as the RNA-dependent RNA polymerase (RdRp), helicase and methyltransferase (Koonin *et al.*, 1992; Perttilä *et al.*, 2013). ORF2 is translated from a subgenomic RNA (sgRNA) and encodes the capsid protein. The non-enveloped virion has icosahedral symmetry and is thought to comprise 180 copies of the capsid protein (i.e. *T* = 3; Mori & Matsuura, 2011). ORF3, which encodes a small accessory protein, overlaps the 5′-terminal region of ORF2 and is translated from the same sgRNA, with the downstream ORF2 initiation codon presumed to be accessed via leaky scanning (Graff *et al.*, 2006). Both genomic and subgenomic RNAs are capped and poly-adenylated. An RNA element occupying codons 35–59 of ORF1 binds the capsid protein *in vitro* and may be involved in genome packaging (Surjit *et al.*, 2004). Two predicted RNA stem–loop structures in the 3′ UTR, and overlapping into the 3′-terminal 13 codons of ORF2, are critical for viral replication (Graff *et al.*, 2005), and may act by binding the viral RdRp (Agrawal *et al.*, 2001). Sequences in the region between ORF1 and ORF2, which are predicted to fold into two RNA stem–loop structures in the antigenome, are also critical for viral replication (Graff *et al.*, 2005b; Cao *et al.*, 2010). These elements are thought to promote sgRNA synthesis from a full-length negative-strand template.

To search for additional functional elements in the HEV genome, we analysed the degree of conservation at synonymous sites in HEV sequence alignments. Regions of enhanced conservation at synonymous sites are indicative of overlapping functional elements such as RNA secondary structures or primary nucleotide sequences with functions in addition to amino acid coding. We identified a number of conserved elements in HEV, with one particularly prominent element being positioned in a central region of ORF2. Here we describe computational and experimental analysis of this element.

## Results and Discussion

The ORF2 sequences of 205 HEV isolates currently available in GenBank were extracted, translated, aligned and back-translated to a nucleotide sequence alignment. The highly divergent avian, bat and rat HEV-like sequences were not included. Next, the alignment was analysed for conservation at ORF2 synonymous sites. This analysis revealed a striking increase in synonymous site conservation in the 110-codon region at the 5′ end of ORF2 that overlaps with ORF3 (Fig. 1b). Selection against synonymous substitutions in ORF2 within this region is expected due to purifying selection acting on ORF3. Similarly, enhanced conservation was observed at the 3′ end of ORF2, corresponding to the previously identified 3′-terminal RNA element. Surprisingly, the analysis revealed an additional region of conservation in a central region of ORF2. The conservation resolves into two distinct highly statistically significant peaks, corresponding (in GenBank RefSeq NC_001434) to codons 384–390 and 402–407 of ORF2 (Fig. 1c, panel 3). High conservation is also apparent in some flanking positions such as codons 376, 417 and 420 (Fig. S1, available in JGV Online).

**Fig. 2.**
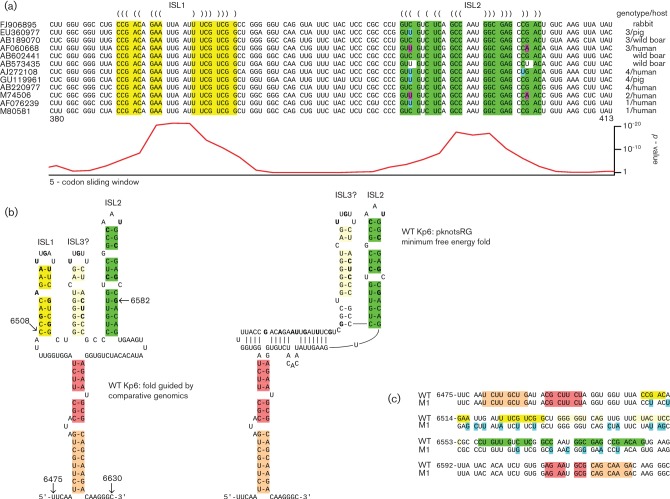
Identification of potential RNA secondary structure in the central conserved region in ORF2. (a) Extracts from representative sequences showing the predicted ISL1 (yellow) and ISL2 (green) stem–loop structures in the conserved region. Predicted base pairings are indicated with parentheses. Single substitutions that preserve the predicted base pairings (e.g. G-C to G-U) are indicated with cyan. Paired substitutions that preserve the predicted base pairings are indicated with pink. GenBank accession numbers are shown at left. Genotype and isolation source species are shown at right. See Johne *et al.* (2012) for a phylogenetic tree. Numbers 380 and 413 below indicate codon positions in ORF2. Note that, in some isolates, ISL1 and ISL2 can be basally extended by, respectively, another one or two base pairings. The corresponding synonymous site conservation *P*-values from Fig. 1 are shown at bottom. See also Fig. S1 for the ISL1, ISL2 and flanking sequences from all 205 ORF2 sequences used. (b) Predicted secondary structure of the ISL1+ISL2 and flanking region from isolate Kp6. Structures ISL1 and ISL2 (left) are based on comparative genomics. In Kp6 and some other isolates, however, the minimum free energy fold (right) contains ISL2 and the peripheral base pairings (indicated with orange/salmon), but ISL1 is disrupted and replaced by a new stem–loop, ISL3. Nucleotides that were altered in the M1 mutant are indicated in bold. Nucleotide coordinates refer to GenBank accession JQ679013. Nucleotides 6508 to 6582 correspond to the region inserted into the HVR. (c) In the M1 mutant, many synonymous mutations (cyan) were introduced within the two regions showing highest conservation (corresponding to ISL1 and ISL2), and additional mutations were introduced to also disrupt the potential ISL3. The colour coding in the WT sequence corresponds to the structure predictions in Fig. 2b. Numbers at left indicate genomic coordinates of the first nucleotide in each line.

Statistically significant peaks in synonymous site conservation are generally indicative of functionally important overlapping elements, either coding or non-coding. In this case, the region of conservation aligns with a short ORF in the −1/+2 reading frame relative to ORF2. However, while short internal ORFs have been shown to be translated via programmed −1 ribosomal frameshifting in a number of other viruses, in this case there was no obvious conserved canonical −1 frameshift site in an appropriate position (Firth & Brierley, 2012). While non-canonical shift sites are certainly possible, an analysis of potential RNA secondary structure suggested a more likely explanation for the conserved region in the centre of ORF2. Secondary structures were predicted by folding the local region of the alignment with Vienna RNA alidot (Hofacker, 2003). The two distinct conservation peaks correspond to two predicted stem–loop structures, hereafter ISL1 and ISL2 (i.e. internal stem–loops 1 and 2; Fig. 2a). Potential base pairing between regions 5′ and 3′ of ISL1+ISL2 may stabilize folding of the two stem–loops (Fig. 2b and Fig. S1). Alignment folding with alidot provided stronger support for structure formation on the positive strand than on the negative strand in this region. This is because some isolates have predicted G : U base pairings, which correspond to C : A non-pairings on the reverse strand (Fig. 2a and Fig. S1).

A similar analysis was performed for ORF1 (185 sequences). This analysis highlighted the previously characterized capsid-binding element near the 5′ end of ORF1, and three much stronger conservation peaks upstream of this element that have not yet been characterized but may represent RNA elements involved in replication, packaging and/or translation of the genomic RNA (Fig. 1c, panel 1). Additionally, a number of more modest conservation peaks were observed at the 3′ end of ORF1 (Fig. 1c, panel 2).

**Fig. 3.**
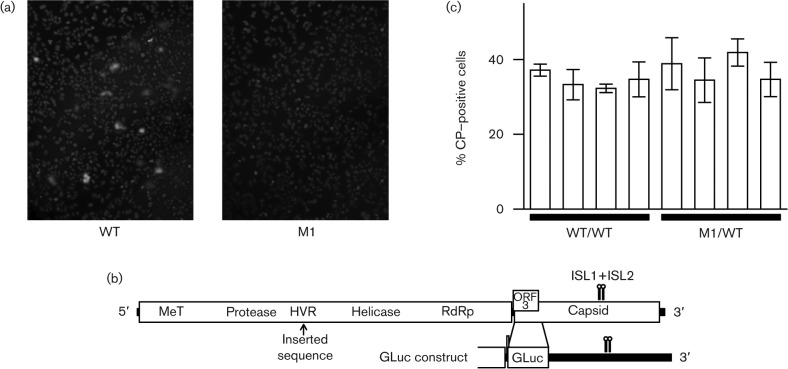
Analysis of ISL1+ISL2 mutants. (a) Representative example of IF microscopy of S10-3 cells stained for capsid protein (green) and nuclei (blue) 4 days after transfection with WT or M1 genomes. (b) Schematic map of WT virus and the GLuc construct indicating the position of the hypervariable region (HVR) and the inserted *Gaussia* luciferase gene relative to ISL1 and ISL2. (c) Flow cytometry of S10-3 cells immunostained for capsid protein 3 days after transfection with WT/WT or M1/WT viruses (four clones of each). Bars indicate the SD of three measurements.

To investigate the role of the ISL1+ISL2 conserved region, we used an infectious cDNA clone of a genotype 3 strain of HEV that grows well in human hepatoma cells (Kp6; GenBank accession JQ679013; Shukla *et al.*, 2012). In Kp6 and some other isolates, the minimum free energy fold (pknotsRG; Reeder *et al.*, 2007) contains ISL2 and a new stem–loop ISL3, but ISL1 is disrupted (Fig. 2b). Whether this occurs *in vivo*, or is simply an artefact of the folding prediction, is uncertain; however, the similarity between ISL1 and ISL3 (in particular within the apical loops) suggests that ISL3 may functionally substitute (or complement) ISL1 in some isolates. A mutant virus, M1, was engineered in which 19 synonymous mutations were introduced into the conserved region to thoroughly disrupt ISL1, ISL2 and the potential ISL3 (Fig. 2c). S10-3 cells (a subclone of the human hepatoma cell line Huh-7) were transfected with *in vitro* transcripts of WT virus or of the M1 virus. At 4 days post-transfection, cells were immunostained for the capsid protein, examined by immunofluorescence microscopy (IF) (Fig. 3a) and the number of stained cells in a representative well of an 8-well chamber slide was counted manually. Since the capsid protein is translated from the sgRNA, viral RNA transcription is required before capsid protein can be produced. In cultures transfected with WT virus, 1209 cells were stained, compared with only 13 faintly stained cells in cultures transfected with the M1 mutant clone. For mock-infected controls, only zero or one possible positive cells were observed per well. Thus the ISL1+ISL2 conserved region is very important for one of the early steps of virus replication (i.e. prior to encapsidation and release). Possibilities include translation of input genomes, genome replication, sgRNA synthesis or sgRNA translation. The M1 mutant was also tested in two other cell lines (human 293 kidney cells and LLC-PK swine kidney cells) and replication was similarly inhibited in each case (data not shown).

ORF1 contains a hypervariable region (HVR; Fig. 3b) that is tolerant of foreign sequence insertions (Shukla *et al.*, 2012). To test whether the mutated M1 sequence was directly detrimental to the virus (e.g. hyper RNase sensitive or difficult to transcribe or translate through), viruses were engineered in which a copy of either the mutated M1 sequence or the corresponding WT sequence (see Fig. 2b, c) was inserted into the HVR. Both these viruses also contained the WT conserved region in its natural position within ORF2 and are correspondingly labelled M1/WT and WT/WT. An additional mutant, WT/M1, containing the WT conserved region in the HVR of the M1 mutant was tested in the same experiment. S10-3 hepatoma cells were transfected and, at 4 days post-transfection, cells were immunostained for the capsid protein, and analysed by IF and by flow cytometry. By IF, the M1/WT and WT/WT mutants appeared to transfect cells and express the capsid protein almost as efficiently as WT virus: the number of positive cells in a representative well of an 8-well slide was 863 for WT, 539 for WT/WT and 778 for M1/WT, compared with 12 for M1. Similarly, flow cytometry analysis revealed no significant difference in the number of capsid-expressing cells between M1/WT (four clones) and WT/WT (four clones) (*t*-test *P* = 0.11; Fig. 3c). Thus the M1 sequence does not appear to be in itself detrimental to virus replication. When the WT/M1 mutant-transfected cells were counted by IF, only 15 cells were positive, a number comparable to the 12 found for M1. Thus the function of the conserved region may be sensitive to its genomic location. However, it is also possible that the attenuated phenotype of WT/M1 was due to the absence of the full ISL1+ISL2 flanking sequences from the cassette inserted into the HVR (Fig. 2b) and/or competing base pairings with flanking HVR sequences (e.g. while pknotsRG predicted that ISL1 and ISL2 would form, RNAfold predicted only ISL1).

**Fig. 4.**
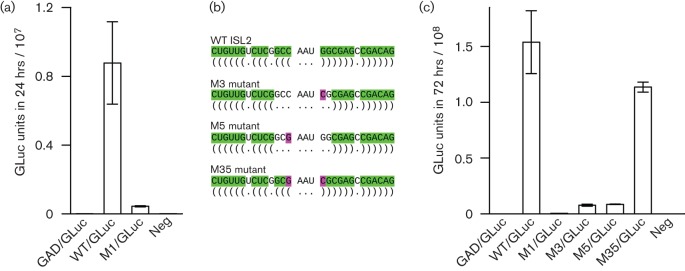
Analysis of virus genomes expressing luciferase. (a) S10-3 cells were transfected with the indicated virus genomes expressing *Gaussia* luciferase and aliquots of medium were tested for luciferase activity at 2 days post-transfection, at which point the harvested medium had been on the cells for 24 h. Bars indicate the SD of three transfections, representing the mean of three aliquots per transfection. WT and M1 refer to the WT and mutant sequences in Fig. 2(c) and GAD is a polymerase mutant. (b) Schematic diagram indicating single substitutions (pink) that, separately, are predicted to disrupt stem–loop ISL2 (M3 and M5 mutants) and, when combined, are predicted to restore the stem–loop structure but with a reversed apical base pairing (M35 mutant). (c) S10-3 cells were transfected with the indicated virus genomes expressing luciferase and aliquots of medium harvested at 5 days post-transfection were assayed for luciferase activity, at which point the harvested medium had been on the cells for 72 h. Bars indicate the SD of three transfections, representing the mean of three aliquots per transfection.

Next, we replaced the 5′-proximal 126 codons of ORF2 with the *Gaussia* luciferase gene (*gluc*), as described previously (Shukla *et al.*, 2012). Translation of *gluc* starts with the ORF2 AUG initiation codon and terminates upstream of the ISL1+ISL2 conserved region (Fig. 3b). *Gaussia* luciferase (GLuc) is expected to be quantitatively excreted into the medium. WT/GLuc is expected to be translated and replicated within cells, and produce sgRNAs, but capsid proteins and therefore infectious virions are not produced (Shukla *et al.*, 2012). Besides WT/GLuc and M1/GLuc virus genomes, we also prepared a polymerase mutant control, GAD/GLuc, in which the polymerase active site GDD was mutated to GAD to prevent any replication. S10-3 hepatoma cells were transfected in triplicate with WT/GLuc, M1/GLuc or GAD/GLuc constructs and aliquots of medium were tested for luciferase activity at 2 days post-transfection. Mean luciferase activity for the M1/GLuc mutant was 20-fold lower than that of WT/GLuc and, as expected, luciferase activity for the polymerase mutant was negligible (Fig. 4a).

To investigate the predicted stem–loop nature of the element, we prepared three new mutant GLuc virus genomes, M3/GLuc (G6574C), M5/GLuc (C6570G) and M35/GLuc (G6574C, C6570G; nucleotide coordinates are relative to JQ679013; Fig. 4b). The M3 and M5 mutations are predicted to disrupt ISL2, while the M35 mutations are predicted to restore the ISL2 secondary structure but with the apical C : G base pairing switched to a G : C base pairing. (We chose to investigate ISL2 because of the potential redundancy between ISL1 and ISL3 in Kp6.) Since the ISL1+ISL2 region is not translated in the GLuc virus (Fig. 3b) these mutations do not alter any amino acid sequences. S10-3 hepatoma cells were transfected in triplicate with each mutant, alongside WT/GLuc, M1/GLuc and GAD/GLuc, and GLuc expression was measured at 5 days post-transfection (Fig. 4c). GLuc expression for GAD/GLuc and M1/GLuc was minimal and, for the M3 and M5 mutants, GLuc expression was 18-to 20-fold lower than WT. On the other hand, GLuc expression for the M35 mutant was only slightly lower than for WT (1.35-fold). The luciferase gene was also moved from ORF2 into the ORF3 reading frame in WT/GLuc and the M3 mutant (G6574C) to verify that expression of ORF3 was affected in parallel with that of ORF2. At day 3 post-transfection, GLuc expression from ORF3 of the mutant was 16-fold lower than that of the WT (data not shown), a difference consistent with the 18- to 20-fold decrease observed when GLuc was expressed from ORF2 of M1 or M3 as seen in Fig. 4 (a, c). These data suggest that correct formation of the ISL2 structure is integral to the function of the ISL1+ISL2 region.

Since ribosomes do not translate through the conserved region in the GLuc constructs, these results would also appear to rule out any role for the conserved region in modulating elongating ribosomes (e.g. stimulating ribosomal frameshifting or altering translational speed to promote correct capsid folding). When the WT and M1 GLuc-expressing sgRNA sequences were fused into CMV vectors and expressed independently, both constructs produced similar amounts of luciferase (2.3±0.4×10^8^ and 2.7±0.8×10^8^ GLuc units in 24 h for WT and M1, respectively; *n* = 3; *t*-test *P* = 0.47; co-transfected ORF2-expressing controls showed no significant difference in average transfection efficiency – 167±14 and 164±68 stained cells per well for WT and M1). This indicates that the mutations had little effect on sgRNA translation or RNA stability. However, attempts to quantify sgRNA synthesis by Taqman RT-PCR failed because of a very high background due to transfecting genomic RNA.

Our results demonstrate that a central conserved region in ORF2 is important for HEV replication. One possibility is that the element plays a role in viral RNA synthesis. Since sgRNA transcription is thought to occur from the full-length negative-strand RNA, the bioinformatic observation that the structure seems more likely to be relevant on the positive strand argues against a direct role in sgRNA synthesis. However, the number of positive-strand G : U base pairings in different isolates that could interfere with formation of the structure on the negative strand is small, especially in ISL1 and the central region of ISL2 (Fig. S1), so the structure may well be active on the negative strand. Alternatively, the structure may play a role in negative-strand synthesis. It is also possible that the conserved region may have some quite different function. Our bioinformatic analysis suggests at least one functionally important aspect of the conserved region is the presence of two predicted stem–loops, ISL1 and ISL2. Analysis of mutants M3, M5 and M35 supports the ISL2 prediction. On the positive strand, the two stem–loops each have an unpaired bulge nucleotide (usually a purine) in the 5′ half of the stem, separated by three base-pairs from the apical loop (Fig. 2a). Moreover, there is a conserved AU at the 3′ end of the loop. These similarities between the two stem–loops, and the intermediate phenotype of the M3 and M5 mutants (in which ISL2 but not ISL1 is disrupted), may suggest a partially redundant protein-binding site.

In other RNA viruses, internal RNA elements play a variety of roles in RNA replication, packaging, subgenomic RNA synthesis and translation (reviewed by Miller & White 2006; Liu *et al.*, 2009; Pathak *et al.*, 2011; Sztuba-Solińska *et al.*, 2011; Firth & Brierley, 2012). Translation-enhancing RNA elements are commonly found near the 5′ ends of coding sequences (e.g. Frolov & Schlesinger, 1996), but internal elements can also modulate translation, e.g. by stimulating ribosomal frameshifting (e.g. Chung *et al.*, 2010). Many internal elements are important for RNA replication; examples include stem–loop structures in alphanodaviruses (Van Wynsberghe & Ahlquist, 2009), the CREs (*cis*-active RNA elements) of picornaviruses (reviewed by Steil & Barton, 2009) and the IREs (internal replication elements) of tombusviruses (Nicholson *et al.*, 2012). Such elements can play roles in recruiting the RNA to the site of replication, interaction with the replication complex and (in picornaviruses) uridylylation of a VPg protein to prime RNA synthesis. Packaging signals in influenza A virus overlap the 5′ and 3′ termini of coding sequences (reviewed by Hutchinson *et al.*, 2010), while in the alphaviuses they are embedded within the coding sequence (Kim *et al.*, 2011). However, internal RNA structures can also play other roles (e.g. the poliovirus ciRNA inhibitor of RNase L; Townsend *et al.*, 2008). Clearly further research is required to establish the precise role of the ISL1+ISL2 element in HEV but, in the meantime, it is important for the community to be aware of the existence of this element. A commonly used technique to analyse HEV growth kinetics involves replacement of part of the capsid-encoding sequence with a luciferase or other reporter gene (see above). It will now be important to ensure that such sequence exchanges do not unintentionally remove or disrupt the RNA element.

## Methods

### 

#### Computational analysis.

Virus sequences were obtained from GenBank most recently on 25 April 2012. HEV nucleotide sequences with coverage of either ORF1 or ORF2 (or both) were identified by applying NCBI TBLASTN (Altschul *et al.*, 1990) to the ORF1 and ORF2 amino acid sequences from the HEV reference sequence NC_001434. In total, 205 ORF2 and 185 ORF1 sequences were retrieved. Within each ORF, sequences were translated, aligned and back-translated to nucleotide sequence alignments using EMBOSS and clustal (Rice *et al.*, 2000; Larkin *et al.*, 2007). The synonymous site conservation statistic was calculated as described previously (Firth *et al.*, 2011).

#### Plasmids.

The p6 infectious plasmid (GenBank accession JQ679013) of the genotype 3, cell-culture-adapted Kernow-C1 virus served as WT and as the parent for all mutants. Nucleotides 79 to 633 encoding the *Gaussia* luciferase gene in the pGLuc basic vector purchased from New England Biolabs were amplified by PCR and inserted into the p6 plasmid between nucleotides 5361 and 5736; this deleted part of the ORF2 gene. Translation of the luciferase gene initiated with the AUG codon of ORF2 and terminated at a UAA codon at the end of the luciferase gene (Shukla *et al.*, 2012). Nucleotides 6508 to 6582 of p6 or M1 were amplified by PCR and inserted into the HVR of p6 or M1 between nucleotides 2243 and 2244 to yield WT/WT, M1/WT and WT/M1. The subgenomic region (from nucleotide 5339 through the poly A region) of p6/GLuc and M1/GLuc were amplified by PCR and cloned into the CMV vector pcDNA 3.3-TOPO (Invitrogen). The sequence of each HEV construct was determined to verify that unwanted mutations had not been introduced.

#### Cell culture.

S10-3 cells, an in-house derived subclone of Huh7 human hepatoma cells, were propagated in Dulbecco’s modified Eagle’s medium supplemented with l-glutamine, penicillin-streptomycin, gentamicin and 10 % FBS (Ultra-Low immunoglobulin G from Invitrogen). Cell stocks were maintained at 37 °C and transfected cultures were maintained at 34.5 °C.

#### Transfection with capped transcripts synthesized *in vitro*.

Virus plasmids were linearized at a unique 3′-terminal *Mlu*I site and 2.5 µg linearized plasmid (1 µg µl^−1^ water) was added per 22.5 µl T7 Riboprobe *in vitro* transcription system (Promega) containing Anti-Reverse Cap Analogue (Ambion). After incubation of the mixture at 37 °C for 90 min, 2 µl was electrophoresed on a 1 % agarose gel to monitor RNA integrity and quantity; the remaining 23 µl was directly mixed with 1 ml Opti-MEM (Gibco) containing 20 µl DMRIE-C (Invitrogen), and the entire mixture was added to a drained monolayer of S10-3 hepatoma cells at ~50 % confluency in a T25 cell culture flask. After incubation for 5 h at 34.5 °C in a CO_2_ incubator, the transfection mixture was replaced with culture medium and incubation was continued at 34.5 °C. For immunofluorescence microscopy (IF), cells were trypsinized and an aliquot was placed on an 8-well chamber slide at 34.5 °C to allow cells to reattach prior to fixation. For luciferase assays, medium was collected, filtered through a 0.45 µm-pore-size Millex HV filter (Millipore) and frozen at −80 °C until tested. Since transfection levels vary considerably from experiment to experiment, only numerical results from the same experiment are compared. However, the conclusions were all confirmed in two or more experiments or with two or more plasmid clones.

#### Transfection with CMV vectors.

Transfection with Lipofectamine 2000 (Invitrogen) was performed according to the manufacturer’s directions for plasmid DNA. M1 and WT CMV plasmids (1.5 µg) expressing GLuc were mixed with 0.4 µg control CMV plasmid expressing HEV ORF2 in 250 µl Opti-MEM and added to 250 µl Opti-MEM containing 5 µl Lipofectamine 2000. Three independent reactions were set up for each GLuc plasmid. After incubation at room temperature for 20 min, each mixture was added to one well of a 6-well tissue culture dish containing S10-3 cells (~95 % confluent) in 2 ml Opti-MEM with 10 % FBS and no antibiotics. Cells were incubated at 37 °C for 24 h and medium was collected from each well, filtered through a 0.45 µm-pore-size Millex HV filter (Millipore) and tested for luciferase activity. Samples from each well were diluted 1 : 1000 and read in triplicate and the mean was used to calculate the mean, SD and *P*-value for the three independent samples. The cells in each well were trypsinized, replated on 8-well chamber slides and incubated at 37 °C for another 24 h. Cells were then acetone-fixed and stained for IF as described below. Cells stained for the internal control ORF2 protein were manually counted.

#### Luciferase assay.

*Gaussia* luciferase activity was determined with the *Renilla* luciferase assay system (Promega). Twenty microlitres of harvested medium was added per well of a 96-well black, flat-bottom microplate (Corning), followed by the addition of *Renilla* luciferase assay substrate and the detection of luminescence using a Berthold LB 960 Centro microplate luminometer. Samples were assayed in triplicate and read sequentially. A standard curve was generated by testing 10-fold serial dilutions of culture medium containing high levels of luciferase activity, and test samples producing values above the linear range were diluted with medium and reassayed until results were in the linear range.

#### Immunofluorescence and flow cytometry.

For IF, cell monolayers on 8-well Lab-Tek IICC^2^ chamber slides were fixed with acetone, washed with PBS and overlaid with a mixture of HEV ORF2-specific hyperimmune serum from an HEV-infected chimpanzee (chimp 1313) and rabbit anti-ORF3 C-terminal peptide antibody. Secondary antibodies (Molecular Probes) were a mixture of Alexa Fluor488-conjugated goat anti-human IgG and Alexa Fluor5 68-conjugated goat anti-rabbit IgG. Stained cells were overlaid with Vectashield mounting medium with DAPI (4′,6′-diamidino-2-phenylindole; Vector Laboratories) and visualized at ×40 with a Zeiss Axioscope 2 Plus fluorescence microscope. The percentage of cells stained for ORF2 protein was estimated relative to 100 % of cells stained with DAPI. Cells stained for capsid protein were counted manually while scanning the entire well on the chamber slide. For flow cytometry, trypsinized cells were pelleted at 525 ***g***, incubated with 1 ml methanol for 15 min on ice, washed with 5 ml PBS and pelleted again. Cells were resuspended in 100 µl blocking solution (0.5 % skim milk, 0.5 % crystalline BSA and 0.1 % Triton X-100 in PBS) at room temperature for 30 min before the addition of 100 µl 2× chimp 1313 serum. After a further 45 min, 5 ml PBS was added, and cells were pelleted and resuspended in 100 µl Alexa Fluor488-conjugated goat anti-human IgG. After 30 min, cells were washed with 5 ml PBS, pelleted and resuspended in 0.3 ml PBS. Cells were analysed with a FACScan flow cytometer (Becton Dickinson) and data were analysed using BD CellQuest software.
